# Histone Deacetylase 11 Contributes to Renal Fibrosis by Repressing KLF15 Transcription

**DOI:** 10.3389/fcell.2020.00235

**Published:** 2020-04-17

**Authors:** Lei Mao, Li Liu, Tao Zhang, Hao Qin, Xiaoyan Wu, Yong Xu

**Affiliations:** ^1^Key Laboratory of Targeted Intervention of Cardiovascular Disease and Collaborative Innovation Center for Cardiovascular Translational Medicine, Department of Pathophysiology, Nanjing Medical University, Nanjing, China; ^2^Department of Geriatric Nephrology, Jiangsu Province Hospital, First Affiliated Hospital of Nanjing Medical University, Nanjing, China; ^3^Institute of Biomedical Research, Liaocheng University, Liaocheng, China; ^4^The Laboratory Center for Basic Medical Sciences, Nanjing Medical University, Nanjing, China

**Keywords:** renal fibrosis, renal tubular epithelial cell, transcriptional regulation, epigenetics, histone deacetylase

## Abstract

Renal fibrosis represents a key pathophysiological process in patients with chronic kidney diseases (CKD) and is typically associated with a poor prognosis. Renal tubular epithelial cells (RTECs), in response to a host of pro-fibrogenic stimuli, can *trans-*differentiate into myofibroblast-like cells and produce extracellular matrix proteins to promote renal fibrosis. In the present study we investigated the role of histone deacetylase 11 (HDAC11) in this process and the underlying mechanism. We report that expression levels of HDAC11 were up-regulated in the kidneys in several different animal models of renal fibrosis. HDAC11 was also up-regulated by treatment of Angiotensin II (Ang II) in cultured RTECs. Consistently, pharmaceutical inhibition with a small-molecule inhibitor of HDAC11 (quisinostat) attenuated unilateral ureteral obstruction (UUO) induced renal fibrosis in mice. Similarly, HDAC11 inhibition by quisinostat or HDAC11 depletion by siRNA blocked Ang II induced pro-fibrogenic response in cultured RTECs. Mechanistically, HDAC11 interacted with activator protein 2 (AP-2α) to repress the transcription of Kruppel-like factor 15 (KLF15). In accordance, KLF15 knockdown antagonized the effect of HDAC11 inhibition or depletion and enabled Ang II to promote fibrogenesis in RTECs. Therefore, we data unveil a novel AP-2α-HDAC11-KLF15 axis that contributes to renal fibrosis.

## Introduction

Chronic kidney disease (CKD) is defined as progressive loss of key renal functions owing to a host of etiological factors including hypertension and diabetes (Coresh, 2017). For a large fraction of patients diagnosed with CKD, a transition to end-stage renal disease (ESRD) and renal failure is inevitable. Without effective long-term renal replacement therapy, prognosis for these patients is poor and mortality rate is high (Liu, 2013). Regardless of its etiology, ESRD is invariably preceded by renal fibrosis, characterized by accumulation of extracellular matrix (ECM) proteins in the renal interstitia, inflammatory infiltrates, proliferation and migration of myofibroblasts, and disruption of normal renal micro-architecture (Zeisberg and Neilson, 2010). Strong evidence suggests that there is a correlation between the severities of renal fibrosis and the outcome of ESRD (Nishitani et al., 2005). Thus, renal fibrosis can be considered as a common end-point for most kidney diseases (Gewin, 2018).

Investigations in the past decade have demonstrated that activated myofibroblasts are the unequivocal effector cell type and driver of renal fibrosis (Falke et al., 2015). Compared to quiescent fibroblasts, myofibroblasts possess a muscle-like contractile phenotype and display much augmented proliferative and migratory ability. There remains great controversy with regard to the origin of myofibroblasts in the fibrotic kidneys. For instance, Humphreys et al. have shown, using the FOXD1-Cre driven tracing mice, that almost 100% of myofibroblasts are derived from pericytes in the unilateral ureteral obstruction (UUO) model and in the ischemia-reperfusion injury model of renal fibrosis (Humphreys et al., 2010). Zeisberg et al. (2007) have shown that up to 35% of myofibroblasts in the kidneys originate from endothelial cells whereas LeBleu et al. have reported that endothelial cells may account for no more than 10% of all myofibroblasts in the kidneys (Zeisberg et al., 2007; LeBleu et al., 2013). This apparent discrepancy stems from the different lineage-tracing tools that have been used in different studies. The evasiveness of “true” myofibroblast identities *in vivo* notwithstanding, many cell types, including renal tubular epithelial cells, fibroblast cells, and endothelial cells, can be induced to adopt a myofibroblast-like phenotype *in vitro* by transforming growth factor (TGF-β), platelet derived growth factor (PDGF-BB), angiotensin II (Ang II), and high glucose (Edeling et al., 2016). Accompanying the transition to myofibroblasts, profound changes in cellular transcriptome is taking place. The underlying epigenetic mechanism, however, is not clear.

The epigenetic machinery plays a key role in regulating mammalian cell transcription. It is generally considered that transcription status can be annotated by different histone modifications. Actively transcribed chromatin is abounded by acetylated histones whereas transcriptionally silenced chromatin is demarcated by low levels of acetylated histones and high levels of methylated H3K9 and H3K27 (Jenuwein and Allis, 2001). Histone acetylation and deacetylation are catalyzed by acetyltransferases and deacetylases, respectively. Traditionally, histone deacetylases (HDACs) fall into one of the four major categories: Class I HDACs, which include HDAC1, HDAC2, HDAC3, and HDAC8; Class II HDACs, which include HDAC4, HDAC5, HDAC6, HDAC7, HDAC9, and HDAC10; Class III HDACs, which include the sirtuin family of NAD^+^-dependent deacetylases; and Class IV HDAC, which contains a sole member HDAC11 (Seto and Yoshida, 2014). Whereas previous studies have demonstrated a role for class I HDACs (Liu et al., 2013; Choi et al., 2016), class II HDACs (Xiong et al., 2019), and class III HDACs (Morigi et al., 2018) in renal fibrosis, little attention has been paid to HDAC11 in this process. In the present study we investigated the role of HDAC11 in renal fibrosis. We report that HDAC11 expression is up-regulated in the fibrotic kidneys in mice and in Ang II-treated tubular epithelial cells *in vitro*. HDAC11 mediates Ang II induced pro-fibrogenic response in tubular epithelial cells by interacting with AP-2α to repress the transcription of KLF15. Therefore, targeting this AP-2α -HDAC11-KLF15 axis may yield novel therapeutic solutions against renal fibrosis.

## Materials and Methods

### Animals

All animal experiments were review and approved by the Intramural Committee on Ethical Conduct of Animal Experiments. To induce renal fibrosis, the unilateral ureteral obstruction (UUO) procedure was performed in the mice. Briefly, a flank incision was made and the left ureter was ligated with silk suture at two points and cut between the ligatures. After the surgery, the animals were injected peritoneally with quisinostat (100 mg/kg, Selleck, Cat# S1096) or vehicle twice a week until the day of sacrifice. The mice were sacrificed 14 days after the surgery. Alternatively, the mice were fed a high-fat diet (HFD, D12492, Research Diets) for 16 weeks as previously described (Xu et al., 2015). In the third model of renal fibrosis, the mice were implanted subcutaneously a minipump (Alzet 2004) that chronically released Ang II (1 μg/kg/min) for 4 weeks.

### Histology

Histological analyses were performed essentially as described (Liu et al., 2018; Zhang et al., 2018b). Paraffin sections were stained with hematoxylin and eosin (Sigma), picrosirius red (Sigma), or Masson’s trichrome (Sigma) according to standard procedures. Pictures were taken using an Olympus IX-70 microscope. Quantifications were performed with Image Pro.

### Cell Culture, Plasmids, Transient Transfection, and Reporter Assay

Immortalized human renal tubular epithelial cells (HK-2, ATCC) and HEK293 cells were maintained in DMEM supplemented with 10% fetal bovine serum (FBS, Hyclone). Primary tubular epithelial cells (Xu et al., 2015), primary podocytes (Murakami et al., 2010), and primary renal fibroblast cells (Kunzel et al., 2019)were isolated as previously described. HDAC11 expression plasmids (Watanabe et al., 2014), HDAC11 promoter-luciferase constructs (Voelter-Mahlknecht et al., 2005), and KLF15 promoter-luciferase constructs (Shao et al., 2018) have been previously described. Small interfering RNAs were purchased from Dhamarcon. Transient transfections were performed with Lipofectamine 2000 (Invitrogen). Cells were harvested 48 h after transfection and reporter activity was measured using a luciferase reporter assay system (Promega) as previously described (Yang et al., 2018).

### Protein Extraction and Western Blot

Before harvesting, cells were washed twice with ice-cold PBS buffer. Cell pellet was obtained by spinning in a refrigerated centrifuge at 2,500 rpm for 10 min. Supernatant was discarded and cells was lysed in ice-cold RIPA buffer (1xPBS, 0.1% SDS, 1% NP-40, 0.5% sodium deoxycholate) supplemented with 100 μg/ml PMSF plus one protease inhibitor tablet (Roche, Mannheim, Germany) per 10 ml RIPA buffer as previously described (Li et al., 2018d; Yang et al., 2019a, b; Zhang et al., 2019). Typically, 50–100 μg of proteins were loaded and separated by 8% PAGE-SDS gel with all-blue protein markers (Bio-Rad). Proteins were transferred to nitrocellulose membranes (Bio-Rad) in a Mini-*Trans-*Blot Cell (Bio-Rad). The membranes were blocked with 5% fat-free milk powder in Tris-buffered saline at room temperature for half an hour and then incubated with the following primary anybodies at 4°C overnight: anti-CTGF (Proteintech, 23936-1), anti-HDAC11 (Abcam, ab166907), anti-α-SMA (Sigma, A5228), anti-KLF15 (Abcam, ab2647), anti-AP-2α (Abcam, ab52222), and anti-β-actin (Sigma, A2228) antibodies.

### RNA Isolation and Real-Time PCR

RNA was extracted with a commercial RNAprep purification kit (Tiangen) as previously described (Shao et al., 2019; Weng et al., 2019). First-strand synthesis was carried out using a HiScript III RT SuperMix (Vazyme). Real-time PCR reactions were performed on an ABI Prism StepOne Plus system with a commercial Sybrgreen kit (Vazyme) using the following primers: mouse *Hdac11*, 5′-TTACAACCGCCACATCTACC-3′ and 5′-GACATTCCTC TC CA CCTTCT C-3′; mouse *Acta2*, 5′-CTGAGCGTGGCTATT CCTTC-3′ and reverse 5′-CTTCTGCATCCTGTCAGCAA-3′; mouse *Col1a1*, 5′-GACGCCATCAAGGTCTACTG-3′ and 5′-AC GGGAATCCA-TCGGTCA-3′; mouse *Col1a2*, 5′-GCCACCAT TGATAGTCTCTCC-3′ and 5′-CACCCCAGCGAAGAACT CATA-3′; mouse *Col3a1*, 5′-GGAACCTGGTTTCTTCTCACC-3′ and 5′-AGGACTGACCAAGGTGGCT-3′; mouse *Tgfb*, 5′-GG AGAGCCCTGGATACCAAC-3′ and 5′-CAACCCAGGTCCT TCCTAAA-3′; mouse *Klf15*, 5′-CCCAATGCCGCCAAACC TAT-3′ and 5′-GAGGTGGCTGCTCTTGGTGTACATC-3′; mouse *Ccl2*, 5′-GAAGGAATGGGTCCAGACAT-3′ and 5′-ACG GGTCAACTTCACATTCA-3′; mouse *Il-1b*, 5′-GCACTACAGG CTCCGAGATGAAC-3′ and 5′-TTGTCGTTGCTTGGTTC TCCTTGT-3′; mouse *Il-6*, 5′-CCAGCTATGAACTCCTTCTC-3′ and 5′-GCTTGTTCCTCACATCTCTC-3′; human *HDAC11*, 5′-ACCCAGACAGGAGGAACCATA-3′ and 5′-TGATGTCC GCATAGGCACAG-3′; human *COL1A1*, 5′-AGGCGAACA GGGCGACAGAG-3′ and 5′-GGCCAGGGAGACCGTTGAGT-3′; human *ACTA2*, 5′-CATCCTCCCTTGAGAAGAGTTA-3′ and 5′-TACATAGTGGTGCCCCCTGATA-3′; human *CTGF*, 5′-GTTTGGCCCAGACCCAACT-3′ and 5′-GGAACA GGCGCTCCACTCT-3′; human *KLF15*, 5′-AGCAAGGACTTG GATGCCTG-3′ and 5′-AGGGCAGGTTCAAGTTGGAG-3′.

### Chromatin Immunoprecipitation

Chromatin Immunoprecipitation (ChIP) assays were performed essentially as described before (Fan et al., 2017, 2019; Li et al., 2017, 2018a,b,c,e, 2019; Yu et al., 2017, 2018; Zeng et al., 2018; Zhang et al., 2018a; Kong et al., 2019a, b; Li and Xu, 2019; Liu et al., 2019a, b). In brief, chromatins were cross-linked with 1% formaldehyde for 15 min at room temperatures. Cells were incubated in lysis buffer (150 mM NaCl, 25 mM Tris pH 7.5, 1% Triton X-100, 0.1% SDS, 0.5% deoxycholate) supplemented with protease inhibitor tablet and PMSF. DNA was fragmented into ∼500 bp pieces using a Diagenode Bioruptor sonicator. For each ChIP reaction, 100 μg of protein were incubated at 4°C overnight with 2 μg of the following antibodies: anti-HDAC11 (Abcam, ab166907), anti-AP-2α (Abcam, ab52222), anti-acetyl H3 (Millipore, 06-599), and anti-acetyl H4 (Millipore, 06-598). For Re-ChIP, immune complexes were eluted with the elution buffer (1% SDS, 100 mM NaCO3), diluted with the Re-ChIP buffer (1% Triton X-100, 2 mM EDTA, 150 mM NaCl, 20 mM Tris pH 8.1), and incubated with a second antibody of interest. Precipitated DNA was amplified with the following primers: *KLF15* promoter #1, 5′-AGCGAGCTGCGGGCGGGCT-3′ and 5′-ACTCTCGGTCCGGCCGGC-3′; *KLF15* promoter #2, 5′-AA GCAAGGAGGTGGCT-3′ and 5′-AAGGCTCGCAGGAGGCT-3′; *KLF15* promoter #3, 5′-AAACCTCCTTAGTCCTG-3′ and 5′-AGTGTCAGATAAATCACTTG-3′; *KLF15* promoter #4, 5′-AGCACCGTCAGCCCACGTG-3′ and 5′-AGTGTCAGATAA ATCACTTG-3′; *KLF15* promoter #5, 5′-AGACCTGCACT GAGAC-3′ and 5′-AGAGGCTTTCTATTC-3′; *GAPDH* promoter, 5′-GGGTTCCTATAAATACGGACTGC-3′ and 5′-CTGGCACTGCACAAGAAGA-3′.

### Immunofluorescence Staining

For immunofluorescence staining, paraffin sections were permeabilized with 0.1% Triton X-100 in PBS for 10 min and then blocked with 5% BSA for 20 min at room temperature followed by incubation with anti-CD3 (BD Biosciences, 1:500) or anti-CD45 (BD Biosciences, 1:500) overnight. The nuclei were counterstained with DAPI (Sigma). 3 slides were stained from each individual mouse and ∼5 fields were counted per slide. The data are presented as the relative number of positive cells/field.

### Statistical Analysis

One-way ANOVA with *post hoc* Scheff’e analyses were performed by SPSS software (IBM SPSS v18.0, Chicago, IL, United States). *P* values less than 0.05 were considered statistically significant.

## Results

### HDAC11 Is Up-Regulated by Pro-fibrogenic Stimuli *in vivo* and *in vitro*

**FIGURE 1:**
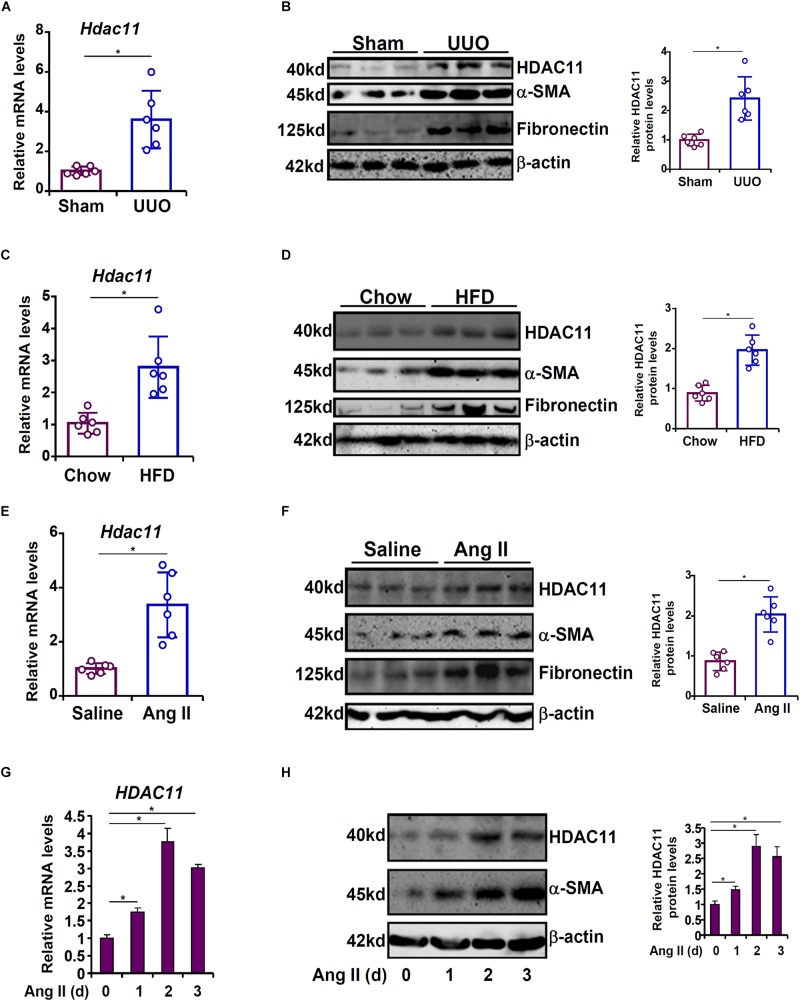
HDAC11 is up-regulated by pro-fibrogenic stimuli *in vivo* and *in vitro*. **(A,B)** C57/BL6 mice were subjected to the UUO procedure or the sham procedure. The mice were sacrificed 2 weeks after the surgery and renal HDAC11 expression was examined by qPCR and Western blot analysis. *N* = 6 mice for each group. **(C,D)** C57/BL6 mice were fed a high-fat diet (HFD) or a control diet for 16 weeks. Renal HDAC11 expression was examined by qPCR and Western blot analysis. *N* = 6 mice for each group. **(E,F)** C57/BL6 mice were implanted with an Ang II minipump as described in section “Materials and Methods.” Renal HDAC11 expression was examined by qPCR and Western blot analysis. *N* = 6 mice for each group. **(G,H)** HK-2 cells were treated with or without Ang II (1 μM) and harvested at indicated time points. HDAC11 expression was examined by qPCR and Western blot analysis.

We first made an attempt to establish a relationship between the expression levels of HDAC11 with renal fibrosis in both animal models and cell models. In the first model, C57/BL6 mice were subjected to the unilateral ureteral obstruction (UUO) procedure. Compared to the sham mice, the UUO mice displayed up-regulation of HDAC11 mRNA paralleling elevation of α-SMA, which correlates with the maturation of myofibroblasts, and fibronectin, a component of the extracellular matrix, in the kidneys (Figures 1A,B). In the second model, C57/BL6 mice were fed with a high-fat diet for 16 weeks to induce renal fibrosis. Quantitative PCR and Western blotting analyses revealed that HDAC11 levels were much higher in the HFD-fed kidneys than in the control-fed kidneys (Figures 1C,D). In the third model, an Ang II-infusion minipump was implanted subcutaneously in C57/BL6 mice for 4 weeks to induce renal fibrosis. Again, it was observed that HDAC11 expression levels were augmented in the Ang II-infused fibrotic kidneys as opposed to the saline-infused kidneys (Figures 1E,F). We also determined whether HDAC11 up-regulation in the fibrotic kidneys occurred universally or only in specific cell compartments. To this end, primary renal tubular epithelial cells, podocytes, and fibroblasts were isolated from the UUO mice or the sham mice. Of interest, HDAC11 was only up-regulated in the tubular epithelial cells isolated from the UUO mice compared to the sham mice but not the podocytes or the fibroblast cells (Supplementary Figure S1). Therefore we focused on the tubular epithelial cells to investigate the role of HDAC11 hereafter.

Angiotensin II (Ang II) has been reported to play a key role promoting renal fibrosis in model animals (Chevalier, 2006; Pandey et al., 2016; Tikoo et al., 2016; Xu et al., 2017). Next, cultured human renal tubular epithelial cells (HK-2) were treated with Ang II. HDAC11 was gradually up-regulated by Ang II stimulation with a similar kinetics as α-SMA: there was a small increase in HDAC11 expression 24 h after the addition of Ang II; HDAC11 expression continued to rise at 48h and declined slightly at 72 h (Figures 1G,H). In order to determine whether Ang II could directly stimulate HDAC11 transcription, a human HDAC11 promoter-luciferase construct was transfected into HK-2 cells. Ang II treatment significantly up-regulated the HDAC11 promoter activity (Supplementary Figure S2); notably, mutation of a conserved NF-κB site site within the HDAC11 promoter abrogated induction by Ang II indicating that NF-κB could potentially mediate the effect of Ang II treatment on HDAC11 transcription. Combined, these data suggest that there might be a positive correlation between HDAC11 and renal fibrosis both *in vivo* and *in vitro*.

### Inhibition of HDAC11 by Quisinostat Attenuates Renal Fibrosis in Mice

**FIGURE 2:**
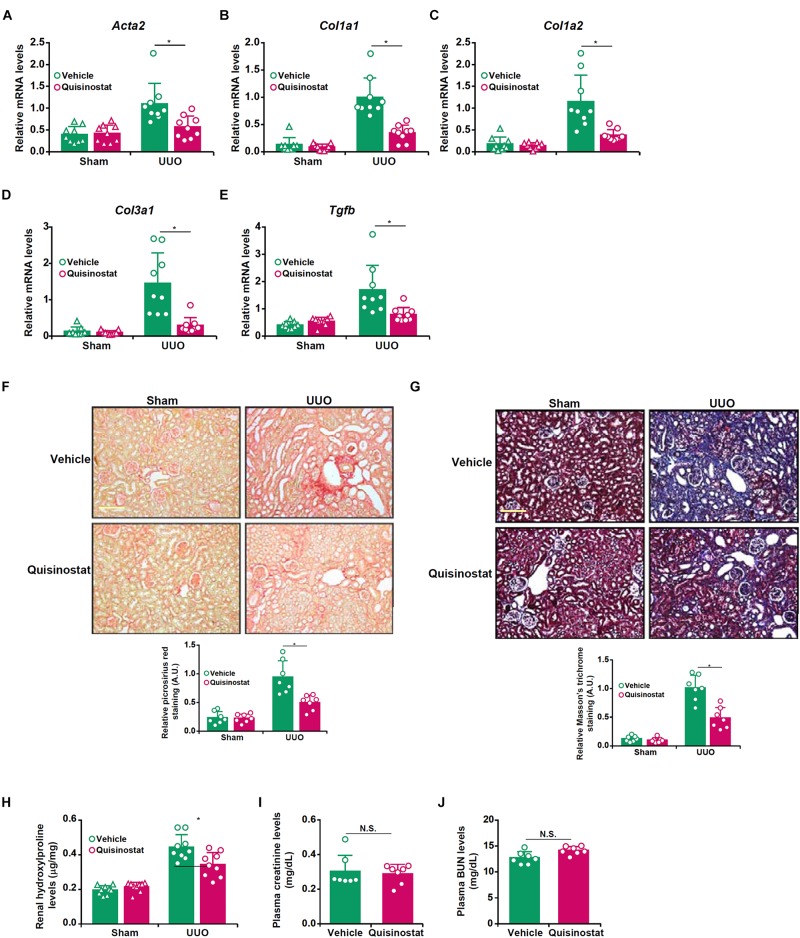
Inhibition of HDAC11 by quisinostat attenuates renal fibrosis in mice. Renal fibrosis was induced in C57/BL6 mice by UUO. After the surgery, the mice were injected with quisinostat or vehicle as described in section “Materials and Methods.” **(A–E)** Expression levels of *Col1a*1 **(A)**, *Col1a*1 **(B)**, *Col3a1* **(C)**, *Acta2* **(D)**, and *Tgfb* **(E)** were examined by qPCR. **(F,G)** Paraffin sections were stained with picrosirius red and Masson’s trichrome. **(H)** Hydroxylproline levels. **(I)** Plasma BUN levels. **(J)** Plasma creatinine levels. *N* = 7∼9 mice for each group.

Based on the observation that HDAC11 was up-regulated in the fibrotic kidneys, we sought to evaluate the effect of pharmaceutical inhibition of HDAC11 on renal fibrosis in the UUO model. To this end, a small-molecule HDAC11 inhibitor quisinostat (Zhou et al., 2017) was administered via peritoneal injection twice a week after the UUO procedure. Quantitative PCR showed that HDAC11 inhibition by quisinostat suppressed the induction of pro-fibrogenic genes such as α-SMA (*Acta2*, Figure 2A), collagen type I (*Col1a1*/*Col1a2*, Figures 2B,C), collagen type III (*Col1a3*, Figure 2D), and transforming growth factor (*Tgfb*, Figure 2E) in the kidneys. Picrosirius red staining (Figure 2F) and Masson’s trichrome staining (Figure 2G) confirmed that renal fibrosis was less extensive in the mice injected with quisinostat than the mice injected with vehicle. Quantification of hydroxylproline levels, as a measurement of total collagen synthesis, in the kidneys showed that administration of quisinostat attenuated renal fibrosis (Figure 2H). Of note, HDAC11 inhibition did not alter UUO-induced impairment of glomerular filtration as evidenced by comparable plasma BUN levels (Figure 2I) and creatinine levels (Figure 2J). We also observed that renal inflammation, as measured by infiltration of CD3^+^ lymphocytes and CD45^+^ leukocytes as well as expression levels of pro-inflammatory mediators in the kidneys, was significantly dampened by quisinostat administration (Supplementary Figure S3).

### HDAC11 Mediates Ang II Induced Pro-fibrogenic Response in HK-2 Cells

**FIGURE 3:**
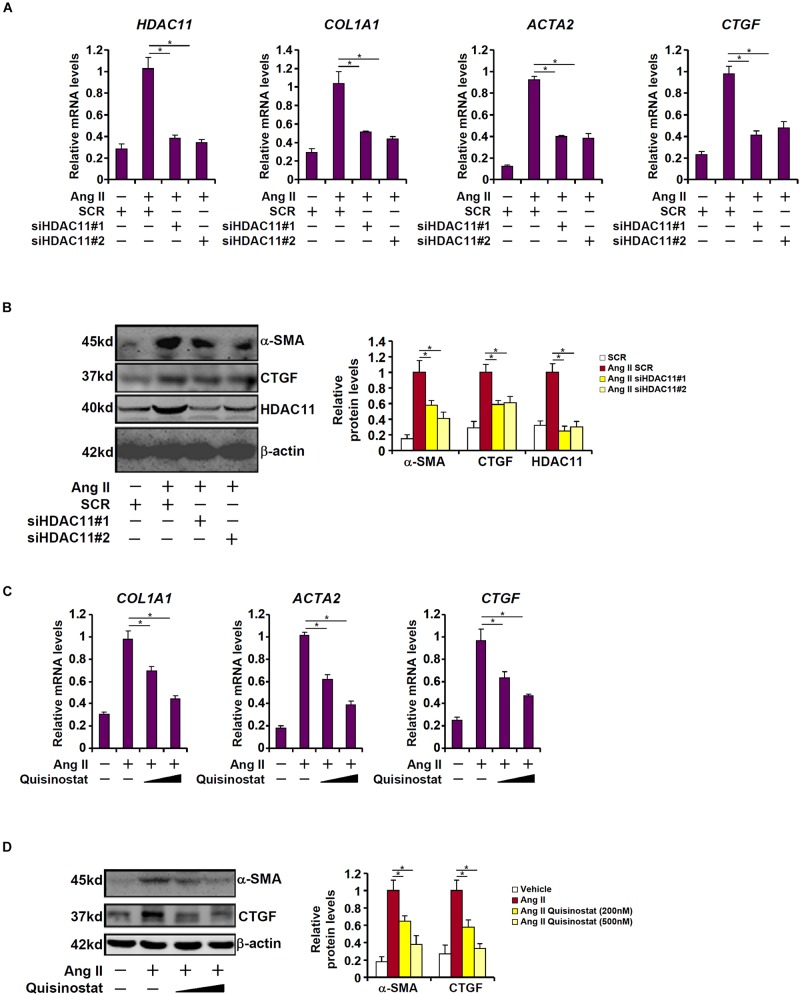
HDAC11 mediates Ang II induced pro-fibrogenic response in HK-2 cells. **(A,B)** HK-2 cells were transfected with small interfering RNAs targeting HDAC11 or scrambled siRNA (SCR) followed by treatment with Ang II (1 μM). Expression levels were examined by qPCR and Western blot analysis. **(C,D)** HK-2 cells were treated with Ang II (1 μM) in the presence or absence of quisinostat (200 nM, 500 nM). Expression levels were examined by qPCR and Western blot analysis.

We next examined the effect of HDAC11 deletion or inhibition on Ang II induced pro-fibrogenic response in HK-2 cells. Exposure of HK-2 cells markedly stimulated the expression of collagen type I, α-SMA, and CTGF as expected; HDAC11 knockdown by two separate pairs of siRNAs abrogated the induction of these pro-fibrogenic genes (Figures 3A,B). Alternatively, co-treatment with quisinostat, the HDAC11 inhibitor, suppressed Ang II-induced pro-fibrogenic response in a dose-dependent manner (Figures 3C,D).

### HDAC11 Is Essential for Ang II Induced KLF15 Repression

**FIGURE 4:**
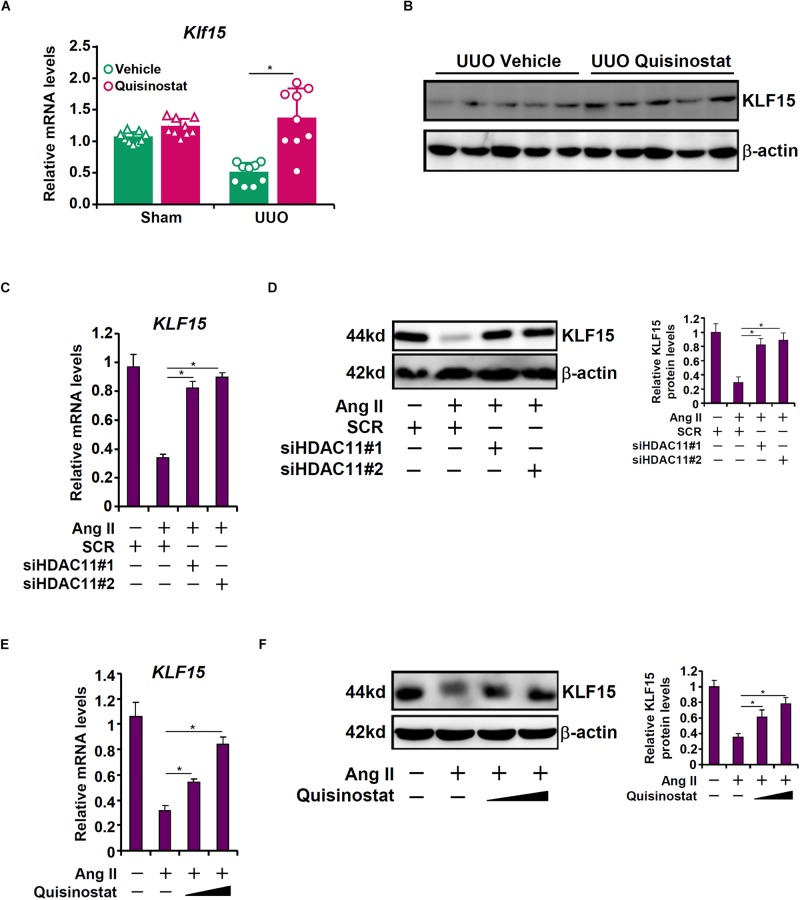
HDAC11 is essential for Ang II induced KLF15 repression. **(A,B)** Renal fibrosis was induced in C57/BL6 mice by UUO. After the surgery, the mice were injected with quisinostat or vehicle as described in section “Materials and Methods.” Renal KLF15 expression was examined by qPCR and Western blot analysis. **(C,D)** HK-2 cells were transfected with small interfering RNAs targeting HDAC11 or scrambled sIRNA (SCR) followed by treatment with Ang II (1 μM). KLF15 expression was examined by qPCR and Western blot analysis. **(E,F)** HK-2 cells were treated with Ang II (1 μM) in the presence or absence of quisinostat (200 nM, 500 nM). KLF15 expression was examined by qPCR and Western blot analysis.

Kruppel-like factor 15 (KLF15) is a transcription factor that has been shown to suppress UUO (Gu et al., 2017a) and Ang II (Gu et al., 2017b) induced renal fibrosis in mice. Of note, KLF15 expression was down-regulated in the UUO kidneys compared to the sham kidneys, which was alleviated by quisinostat administration (Figures 4A,B). We then hypothesized that HDAC11 might mediate Ang II induced repression of KLF15 in RTECs. Ang II treatment led to a reduction of KLF15 mRNA (Figure 4C) and protein (Figure 4D) levels; HDAC11 knockdown largely normalized KLF15 expression. Similarly, HDAC11 inhibition by quisinostat dose-dependently antagonized repression of KLF15 expression by Ang II treatment (Figures 4E,F).

**FIGURE 5:**
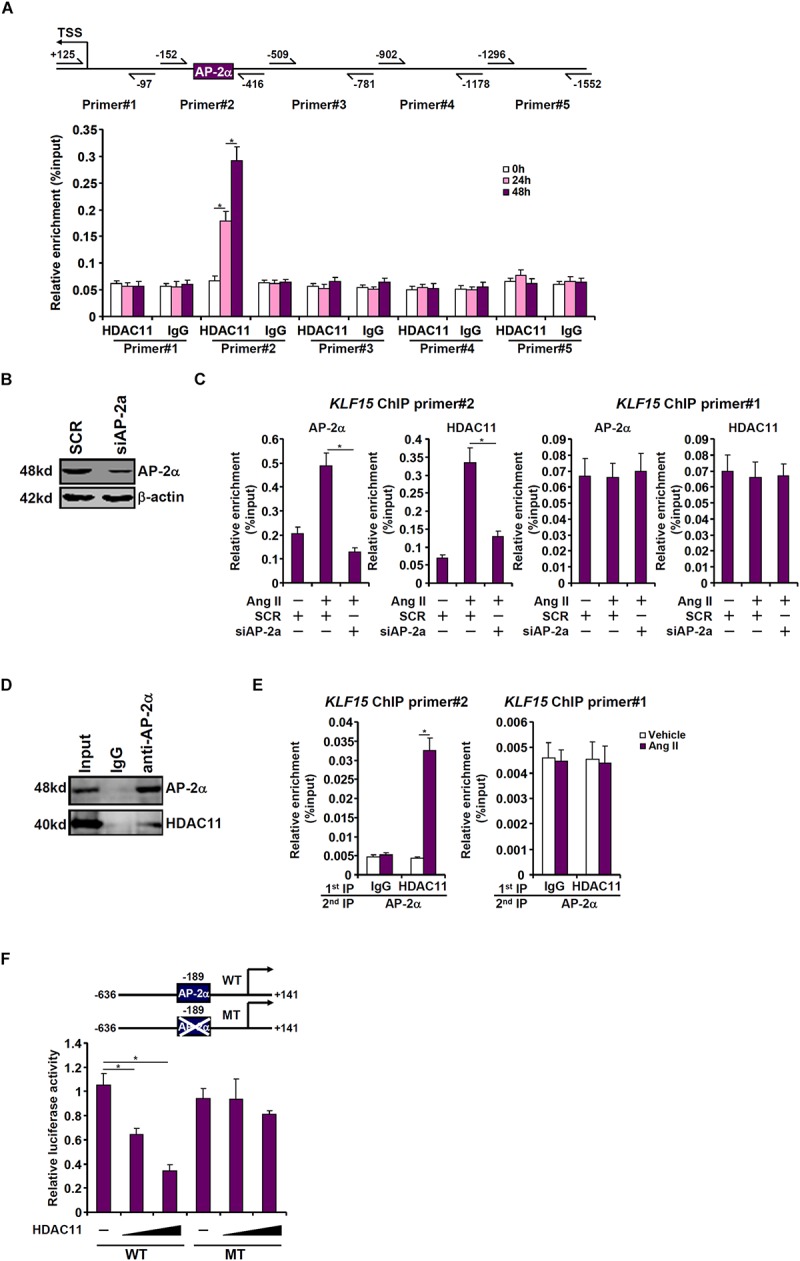
HDAC11 interacts with AP-2α to repress KLF15 transcription. **(A)** HK-2 cells were treated with Ang II (1 μM). The cells were harvested at indicated time points and ChIP assays were performed with an anti-HDAC11 antibody or IgG. **(B,C)** HK-2 cells were transfected with siRNA targeting AP-2α or SCR followed by treatment with Ang II (1 μM) for 48 h. ChIP assays were performed with an anti-HDAC11 antibody. **(D)** HK-2 cells were treated with Ang II (1 μM) for 48 h. Nuclear proteins were extracted and immunoprecipitation was performed with indicated antibodies. **(E)** HK-2 cells were treated with or without Ang II (1 μM) for 48 h. Re-ChIP assay was performed with indicated antibodies. **(F)** Wild type or mutant KLF15 promoter-luciferase construct was transfected into HK-2 cells with or without HDAC11. Luciferase activities were normalized by GFP fluorescence and protein concentration.

To determine the region within the KLF15 promoter to which HDAC11 might bind, ChIP assays were performed with an anti-HDAC11 antibody or a control IgG. Precipitated DNA was amplified by primers that span ∼1.5 kb of the proximal KLF15 promoter. As shown in Figure 5A, Ang II treatment specifically stimulated the recruitment of HDAC11 to a region within the proximal KLF15 promoter (−152/−416) that contains a binding site for the transcriptional repressor AP-2α. Small interfering RNA targeting AP-2a was used to deplete endogenous AP-2α to test the possibility that AP-2α might be responsible for recruiting HDAC11 to the KLF15 promoter (Figure 5B). ChIP assays revealed that Ang II treatment significantly augmented the occupancies of both AP-2α and HDAC11 on the KLF15 promoter; AP-2α knockdown, however, abolished the binding of both (Figure 5C). Additional experiments were performed to confirm the interaction between HDAC11 and AP-2α. An anti-AP-2α antibody simultaneously precipitated both AP-2α and HDAC11 from nuclear lysates extracted from HK-2 cells, suggesting that AP-2α and HDAC11 may form a complex (Figure 5D). Further, Re-ChIP assay showed that Ang II treatment strongly enhanced the interaction between AP-2α and HDAC11 on the KLF15 promoter (Figure 5E). Functionally, over-expression of HDAC11 dose-dependently repressed the KLF15 promoter activity, which was blunted by the mutation of the AP-2α site (Figure 5F). Together, these data seem to support a role for the AP-2α-HDAC11 complex in mediating Ang II induced KLF15 repression in tubular epithelial cells.

### HDAC11 Promotes Pro-fibrogenic Response Through KLF15 in HK-2 Cells

**FIGURE 6:**
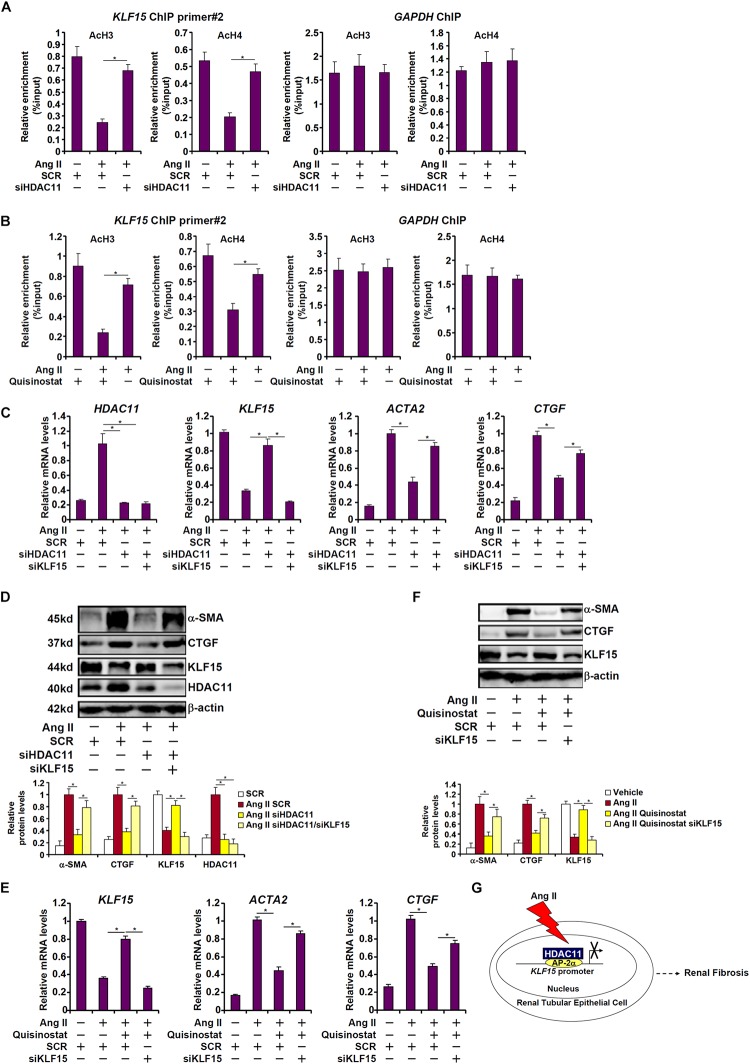
HDAC11 promotes pro-fibrogenic response through KLF15 in HK-2 cells. **(A)** HK-2 cells were transfected with small interfering RNAs targeting HDAC11 or scrambled sIRNA (SCR) followed by treatment with Ang II (1 μM). ChIP assays were performed with anti-acetyl H3 and anti-acetyl H4 antibodies. **(B)** HK-2 cells were treated with Ang II (1 μM) in the presence or absence of quisinostat (500 nM). ChIP assays were performed with anti-acetyl H3 and anti-acetyl H4 antibodies. **(C,D)** HK-2 cells were transfected with small interfering RNAs targeting HDAC11, KLF15, or scrambled siRNA (SCR) followed by treatment with Ang II (1 μM). Expression levels were examined by qPCR and Western blot analysis. **(E,F)** HK-2 cells were transfected with small interfering RNAs targeting KLF15, or scrambled siRNA (SCR) followed by treatment with Ang II (1 μM) and/or quisinostat (500 nM). Expression levels were examined by qPCR and Western blot analysis. **(G)** A schematic model.

Histone deacetylase 11 is an atypical (class IV) histone deacetylase. We examined the effect of HDAC1 depletion/inhibition on hisone acetylation surrounding the KLF15 promoter. When HK-2 cells were exposed to Ang II, there was a simultaneous loss of acetyl H3 and acetyl H4 from the KLF15 promoter, but not from the GAPDH promoter, consistent with repression of KLF15 transcription (Figures 6A,B). HDAC11 depletion by siRNA (Figure 6A) or HDAC11 inhibition by quisinostat (Figure 6B) largely restored histone acetylation surrounding the KLF15 promoter, indicating that HDAC11 likely contributes to KLF15 repression by modulating histone acetylation levels. We then asked whether the ability of HDAC11 to promote Ang II induced pro-fibrogenic response relies on KLF15. As shown in Figures 6C,D, whereas HDAC11 knockdown suppressed induction of pro-fibrogenic gene expression by Ang II, simultaneous depletion of HDAC11 and KLF15 restored the pro-fibrogenic response induced by Ang II. Similarly, the loss of KLF15 antagonized the effect of quisinostat and enabled Ang II to promote fibrogenesis in HK-2 cells (Figures 6E,F). We therefore conclude that KLF15 may be the primary target of HDAC11 during Ang II induced pro-fibrogenic response in tubular epithelial cells.

## Discussion

Epigenetic regulation of renal fibrosis is one of the most actively investigated areas in deciphering the pathogenesis of and finding the solutions for end-stage renal diseases (Tampe and Zeisberg, 2014; Kato and Natarajan, 2019). Here we report that the histone deacetylase HDAC11 promotes renal fibrosis by epigenetically repressing the transcription of KLF15, an anti-fibrogenic factor. Inhibition of HDAC11 with a small-molecule compound quisinostat attenuates UUO-induced renal fibrosis in mice. Therefore, our data provide the proof-of-concept for targeting HDAC11 as a potential therapeutic solution against renal fibrosis. However, there are a few caveats that deserve further attention regarding the current working model (Figure 6G). First, we focused our investigation on renal tubular epithelial cells. It has been reported that other cell types, including fibroblasts, endothelial cells, and myeloid cells, can contribute to renal fibrosis (Zeisberg and Neilson, 2010; LeBleu et al., 2013; Mack and Yanagita, 2015); relatively little is known regarding the role of HDAC11 in these cells. HDAC11 plays a regulatory role in myeloid cells (Yanginlar and Logie, 2018). For instance, Wang et al. (2011) have previously reported that HDAC11 represses the transcription of IL-10 in macrophages. Administration of IL-10, coincidently, can suppress renal fibrosis in rats subjected to 5/6 nephrectomy (Mu et al., 2005). In addition, HDAC11 can modulate the function of myeloid derived suppressor cells (MDSCs), a heterogeneous population of immune cells specialized in the suppression of T lymphocyte function (Sahakian et al., 2015). Hsieh et al. (2018) have shown that administration of MDSCs ameliorates renal fibrosis in diabetic mice. These observations collectively appear to suggest that HDAC11 may contribute to renal fibrosis by regulating the immune microenvironment in the kidneys. Second, HDACs, in addition to removing the acetyl group from histones, can also de-acetylate non-histone proteins. The class III HDAC SIRT1 mitigates renal fibrosis, in part, by deacetylating and deactivating SMAD3, a key transcription factor involved in fibrogenesis (Li et al., 2010). HDAC1 and HDAC2 can promote the deacetylation of STAT1, which prevents its binding to and inhibition of NF-κB allowing the latter to stimulate a pro-fibrogenic transcription program in mesangial cells (Kumar et al., 2017). The acetylation status and thus activity of STAT3, a pro-fibrogenic transcription factor, can also be modulated by HDACs during renal fibrosis (Ni et al., 2014). Since specific non-histone substrates for HDAC11 have yet to be identified, profiling the HDAC11 interactome in the kidneys may provide novel insight into its mode of action in the context of renal fibrosis.

There is growing body of evidence that supports an anti-fibrotic role of KLF15 in the kidneys. Mei and colleagues were among the first to report that KLF15 levels were decreased in the kidneys of rats in a model of chronic renal disease (CKD) and that KLF15 deletion sensitized the mice to the development of renal fibrosis (Gao et al., 2011; Gu et al., 2017a). Mechanistically, KLF15 may regulate renal fibrosis by suppressing the ERK/MAPK, the JNK/MAPK, and the Wnt/β-catenin pathways (Gao et al., 2013; Gu et al., 2017a). More recently, Lu et al. have shown that KLF15 may regulate renal fibrosis by modulating activity of the TGF-β downstream mediators SMAD2/3 via its *trans-*activation domain (TAD) (Lu et al., 2019). Although KLF15 levels can be down-regulated by a host of pro-fibrotic stimuli in the kidneys the underlying mechanism remains obscure. We show here that AP-2α recruits HDAC11 to repress KLF15 transcription in tubular epithelial cells. Although a vast majority of the studies conducted so far have portrayed AP-2α as a regulator of lineage specification during embryogenesis and cancer development and progression in adults (Eckert et al., 2005; Kolat et al., 2019), there is indication that AP-2α may play a key role in cellular fibrogenic response. AP-2α can promote epithelial-mesenchymal transition (EMT), a process critical to tissue fibrosis, by up-regulating TGF-β expression (Zhang et al., 2017) and by forming a complex with ZEB1/2, the E-box binding transcriptional regulators of EMT (Dimitrova et al., 2017). Ross et al. (2019) have recently reported that deletion of TFAP2A (the gene encoding AP-2α) significantly attenuates the TGF-β induced maturation of myofibroblasts although the underlying mechanism is unclear. Traditionally, transcription factors are notorious to target in drug development. Recent successes in “drugging” such transcription factors as p53 (Khoo et al., 2014) and c-Myc (Dang et al., 2017) may shed some light on this issue should further evidence present AP-2α and/or KLF15 as a desirable target in the intervention of renal fibrosis.

There are several limitations of the present study that necessitate cautious interpretation of the data within. First, we relied exclusively on quisinostat to evaluate the effect of HDAC11 on renal fibrosis *in vivo*. Quisinostat is not a strictly specific HDAC11 inhibitor because it can, with equivalent potency, target several other HDACs that have been demonstrated to play regulatory roles in renal fibrosis (Arts et al., 2009). Therefore, it remains uncertain whether the anti-fibrotic effects of quisinostat administration are achieved by HDAC11 inhibition. Future studies employing tissue-specific HDAC11 knockout mice (Bagchi et al., 2018) will hopefully provide solid genetic evidence to ascertain the role of HDAC11 in renal fibrosis. Second, the *in vitro* data were based on a single cell model (Ang II treated human tubular epithelial cells), which makes it difficult to reconcile them with the *in vivo* data especially in the light of the recent finding that tubular epithelial cells derived myofibroblasts only constitute a small fraction of the overall population of myofibroblasts in the fibrotic kidneys in mice (LeBleu et al., 2013). Therefore the issue as to whether HDAC11-driven synthesis of pro-fibrogenic molecules in tubular epithelial cells in response to Ang II treatment plays a significant role in the pathogenesis of renal fibrosis *in vivo* needs to be revisited in the future.

In summary, our data suggest that an AP-2α-HDAC11-KLF15 axis is involved in the pathogenesis of renal fibrosis. Small-molecule inhibitors that target this axis may be considered as a potential therapeutic strategy for the treatment of end-stage renal diseases.

## Data Availability Statement

The datasets generated for this study are available on request to the corresponding author.

## Ethics Statement

The animal study was reviewed and approved by the Nanjing Medical University Committee on Ethical Conduct of Animal Experiments.

## Author Contributions

YX conceived the project and wrote the manuscript with inputs from all authors. LM and XW designed the experiments. LM, LL, TZ, HQ, and XW performed the experiments and collected and analyzed the data. TZ and XW provided funding and supervision.

## Conflict of Interest

The authors declare that the research was conducted in the absence of any commercial or financial relationships that could be construed as a potential conflict of interest.
