# Supplementary material for: Histone Deacetylase 11 Contributes to Renal Fibrosis by Repressing KLF15 Transcription
Source: Front Cell Dev Biol. 2020 Apr 17;8:235. doi: 10.3389/fcell.2020.00235 (PMC7180197; doi:10.3389/fcell.2020.00235)
Supplement: Supplementary file 1 [file Table_1.DOCX]

**Mao L et al: Histone deacetylase 11 contributes to renal fibrosis by repressing KLF15 transcription**

**Online supplementary material**

**Fig.S1:** C57B/6 mice were subjected to the UUO procedure or the sham procedure. The mice were sacrificed 14d after the surgery. Primary renal tubular epithelial cells, podocytes, and renal fibroblasts were isolated and HDAC11 expression was examined by qPCR. N=3 mice for each group.

**Fig.S2:** Wild type or NF-κB site mutated HDAC11 promoter-luciferase construct was transfected into HK-2 cells followed by treatment with Ang II (1μM). Luciferase activities were normalized by GFP fluorescence and protein concentration.

**Fig.S3**: Renal fibrosis was induced in C57/BL6 mice by UUO. After the surgery, the mice were injected with quisinostat or vehicle as described in Methods. (A) Expression of pro-inflammatory mediators was examined by qPCR. (B) Infiltration of immune cells was examined by immunofluorescence staining.
